# Development of Strategies for SNP Detection in RNA-Seq Data: Application to Lymphoblastoid Cell Lines and Evaluation Using 1000 Genomes Data

**DOI:** 10.1371/journal.pone.0058815

**Published:** 2013-03-26

**Authors:** Emma M. Quinn, Paul Cormican, Elaine M. Kenny, Matthew Hill, Richard Anney, Michael Gill, Aiden P. Corvin, Derek W. Morris

**Affiliations:** TrinSeq and Neuropsychiatric Genetics Research Group, Department of Psychiatry and Institute of Molecular Medicine, Trinity College Dublin, Dublin, Ireland; The University of Arizona, United States of America

## Abstract

Next-generation RNA sequencing (RNA-seq) maps and analyzes transcriptomes and generates data on sequence variation in expressed genes. There are few reported studies on analysis strategies to maximize the yield of quality RNA-seq SNP data. We evaluated the performance of different SNP-calling methods following alignment to both genome and transcriptome by applying them to RNA-seq data from a HapMap lymphoblastoid cell line sample and comparing results with sequence variation data from 1000 Genomes. We determined that the best method to achieve high specificity and sensitivity, and greatest number of SNP calls, is to remove duplicate sequence reads after alignment to the genome and to call SNPs using SAMtools. The accuracy of SNP calls is dependent on sequence coverage available. In terms of specificity, 89% of RNA-seq SNPs calls were true variants where coverage is >10X. In terms of sensitivity, at >10X coverage 92% of all expected SNPs in expressed exons could be detected. Overall, the results indicate that RNA-seq SNP data are a very useful by-product of sequence-based transcriptome analysis. If RNA-seq is applied to disease tissue samples and assuming that genes carrying mutations relevant to disease biology are being expressed, a very high proportion of these mutations can be detected.

## Introduction

The transcriptome consists of all RNA transcripts, coding or non-coding, expressed within a given cell or tissue. Its annotation and quantification has been the subject of extensive investigation for several decades. Studying the transcriptome in disease tissue can give important insights into the functional properties of specific RNA transcripts and thereby provide a clearer understanding of the underlying disease processes.

Until very recently the predominant means of studying the transcriptome was using hybridisation based methods such as microarrays [1]. These however are not without limitations; difficulties in monitoring the efficiency of probe hybridisation, cross hybridisation as a result of repetitive regions and issues relating to the normalisation of transcript levels in relation to transcript abundance are common. Probe design is inherently based on known sequences therefore limiting the extent of novel gene/transcript and splice discovery that is possible, although tiling microarrays are now available [2].

Next generation sequencing technologies have rapidly changed transcriptome analysis as researchers acknowledge the benefits of RNA sequencing (RNA-seq). This methodology, which allows the direct sequencing of cDNA libraries, allows for more accurate quantification of RNA transcripts in a given cell or tissue [3] but importantly requires no prior sequence knowledge thereby allowing the discovery of new genes, transcripts, alternative splice junctions, fused sequences and novel RNAs [4]. RNA-seq has been used to examine differential gene expression for different genes and tissues [5] but has also been applied to the study of allelic differences in expression [6], [7] transcriptome characterisation [8], [9] analysis of RNA-protein interactions [10] and analysis of alternative splicing [11].

RNA-seq can be performed on RNA extracted from disease tissue or blood directly obtained from an individual. For a large number of disease studies it has become increasingly common to generate lymphoblastoid cell lines (LCLs) for patient samples using EBV transformation of blood lymphocytes. This not only provides an unlimited source of patient DNA but gives researchers a valuable source of RNA to use for gene expression/functional studies [12] and many large-scale LCL repositories now exist. LCLs have been shown to be a reliable source material for SNP genotyping in genomic DNA [13] and studies of genetic variation in gene expression [14]. The Welcome Trust Case Control Consortium have successfully performed genome-wide association studies (GWAS) using SNPs and copy number variation (CNVs) for eight diseases using a common control panel where half of the 3,000 control DNA samples were derived from LCLs [15], [16]. Whilst expression results generated in cell lines should be interpreted with caution, some recent studies have supported the use of human lymphocytes as a good cellular model for gene expression in other tissues [17]–[19] and there is evidence that expression quantitative trait loci detected in LCLs can overlap with those found in relevant tissues [20].

As with transcriptome analysis, array-based genome-wide analysis of DNA sequence variation is being superseded by next-generation sequencing, which offers the opportunity to detect all variants present and not just assay the variants targeted by pre-designed arrays. Whole-genome sequencing is being applied in the 1000 Genomes (http://www.1000genomes.org/) project to expand on resources such as HapMap to include rare variation [21]. This is particularly important for researchers studying complex diseases as, for many disorders, a substantial proportion of their heritability may be a result of rare variants in the form of SNPs, indels or CNVs [22].

While whole genome sequencing remains costly for disease genetics, an intermediate option is exome sequencing [23] on the premise that the majority of disease related mutations are located within coding sequences (approximately 1–2% of the genome). From Mendelian disease we know that mutations causing amino acid changes account for ∼60% of disease mutations [24]. While RNA-seq is primarily considered a method of gene expression analysis, it is also a form of exome sequencing with the capacity to detect sequence variation in those genes that are expressed in the sample. Therefore, a major advantage of RNA-seq is to offer a convergent approach to disease research by providing information for gene expression/characterisation and also coding sequence variation plus potential insight into post translational processes such as RNA editing.

A number of studies have reported on the viability of SNP detection using RNA-seq[7], [25]–[31] but the purpose of this study is to determine the best approach for RNA-seq SNP analysis by evaluating the performance of different alignment strategies and SNP-calling methods in comparison to extensive available online sequence variation data such as 1000 Genomes data. To do this we calculate the specificity and sensitivity of RNA-seq SNP detection. Specificity addresses the question: how likely is a SNP detected by RNA-seq to be a true variant in the DNA sequence? Sensitivity addresses the question: how likely is RNA-seq to detect an expressed SNP if it is present in a transcribed gene? Overall the results indicate that RNA-seq is a very accurate method of SNP detection. Where genes are strongly expressed, a high proportion of coding SNPs will be correctly identified.

## Materials and Methods

### Sample Preparation and RNA Sequencing

Total RNA was extracted from the HapMap CEU lymphoblastoid cell line sample (Coriell Institute for Medical Research) for individual NA12878 using Qiagen’s RNeasy mini kit. Illumina sequencing libraries were prepared according to the Illumina mRNA Sequencing Sample Prep Guide (1004898 Rev. D). Briefly, poly-A-containing molecules were purified and fragmented, followed by double-stranded cDNA synthesis. The resulting double-stranded cDNA was end-repaired before ligation of Illumina-specific adaptors and finally was PCR enriched. Sequencing was carried out on an Illumina Genome Analyzer II to produce three lanes (A–C) of 40 bp single-end reads (lane A = 11,683,367, lane B = 13,980,330 and lane C = 15,120,659 reads) see table S1). Data from lanes A, B and C combined to create the test sample dataset (40,784,356 reads). This RNA-seq data for sample NA12878 has been deposited in the Short Read Archive (http://www.ncbi.nlm.nih.gov/sra; SRA065878). In addition to generating data in-house on NA12878, we accessed RNA-seq data for HapMap samples NA12891 (80,062,322 reads) and NA12892 (80,023,135 reads) from the Short Read Archive (SRR074943, SRR074953). In order to compare these data to our in-house data, we trimmed the reads for these two samples to a length of 40 bp.

### Analysis Strategy and Methods

**Figure 1:**
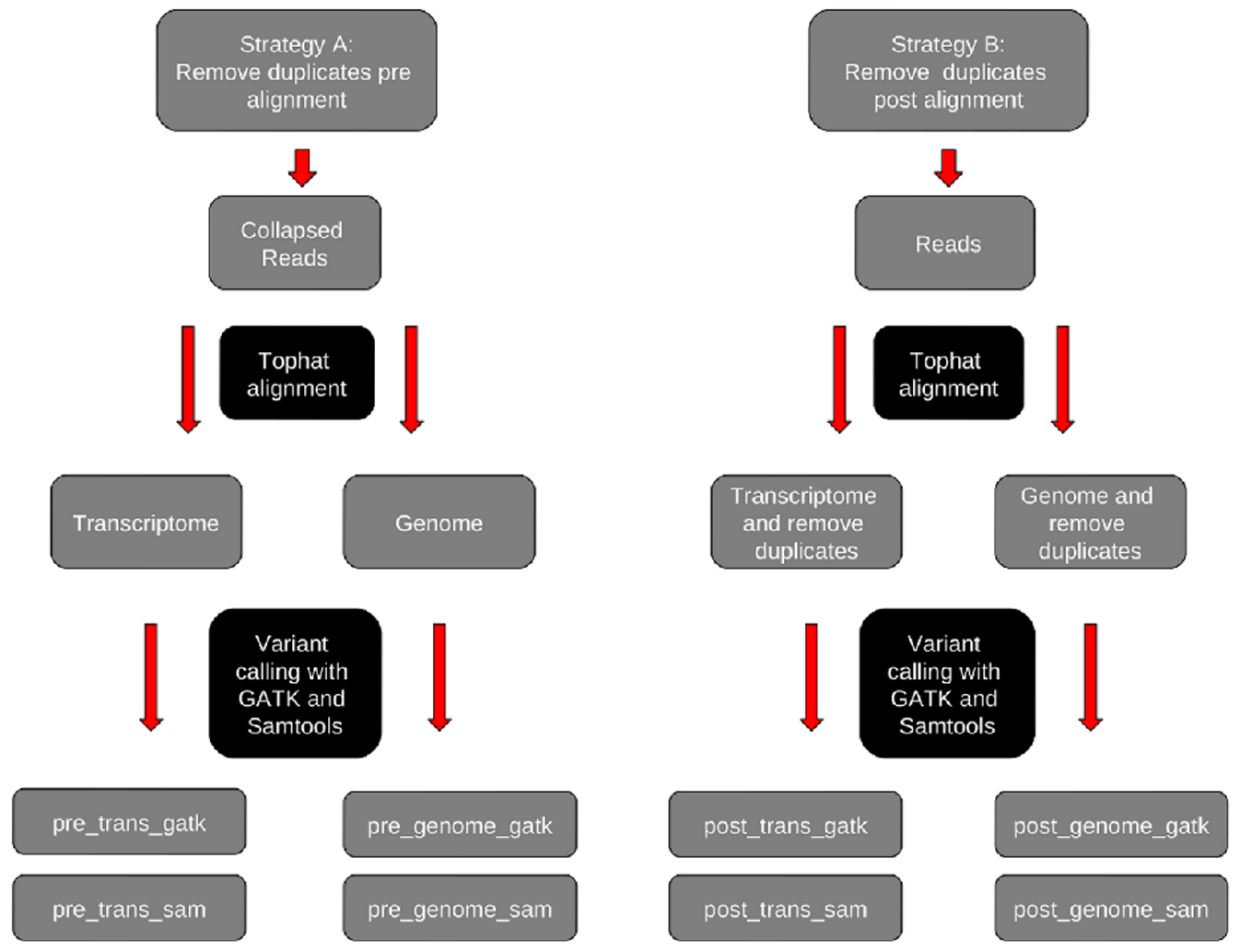
Analysis strategies and methods for RNA-seq SNP detection. This figure outlines the analysis strategies and methods used to identify the best performing methods of RNA-seq SNP detection. We analyzed our data by removing duplicates pre-alignment (strategy A) and post-alignment (strategy B). Reads were aligned to either the genome or the transcriptome and SNP calls generated using SAMtools and GATK. This produced 8 sets of calls for analysis (see table S2a).

Figure 1 gives an overview of the 8 analysis strategies and methods that we employed to identify the best performing methods of RNA-seq SNP detection. Strategies are outlined in detail below but in brief this involved removing duplicate reads pre- and post-alignment to either the genome or transcriptome and the use of two different SNP callers.

### Duplicate Reads

The first decision was if and when to remove identical duplicate reads from analysis. Duplicate reads in sequence data can occur during the PCR/library preparation steps, from sequencing artefacts such as poly-A and poly-N reads, noise in cluster detection and from cDNA fragmentation at the same location in different molecules [32]. This can lead to an exaggeration of coverage levels and impact the accuracy of variant calls. To avoid this, where duplicate reads are detected, only one copy of the read is kept and duplicates are generally removed either pre- or post-alignment. The difference between pre- and post-alignment strategies is as follows: Duplicate reads dropped pre-alignment have exact identical sequence whereas those dropped post-alignment are reads that map to the same position in the genome or transcriptome, i.e. have the same start and end coordinates, but can contain sequence differences internally. There is no gold standard method to deal with duplicates. Removal of duplicates pre-alignment will miss duplicate reads that are derived from the same cDNA fragment but contain sequencing errors, therefore resulting in an underestimation of duplicate reads. Removal of duplicates post-alignment could exclude a read containing the second allele of a SNP and thus will result in loss of valuable information. To determine the best method of dealing with duplicates we analyzed our data by removing duplicates pre-alignment (strategy A) and post-alignment (strategy B) and studied the impact on the number and quality of SNP calls. Table S1 details the number of duplicate reads detected for each sample.

### Alignment and SNP Calling

Tophat version 1.4.1 was selected for alignment of the generated RNA-seq reads with two mismatches allowed per uniquely aligned read. We wanted to compare SNP calls generated when aligning reads to the reference genome compared to a transcriptome-only approach and therefore each sample was aligned using both duplicate read methods to the reference genome (NCBI build 36.1) and to the associated refFlat gene list from UCSC, which represents the transcriptome (see figure 1). In addition, two different publically available SNP calling softwares were used; SAMtools v-1.18 [33] and GATK (Genome analysis toolkit (v-1.0.5506) [34], both of which used in the analyses of data from the 1000 Genomes project(21).

To summarize, eight different variant calling strategies were carried out. In strategy A, identical sequences were removed pre-alignment and the remaining reads were aligned to both a transcriptome reference and a whole genome reference. Two SNP callers were then applied to the alignment files generated, resulting in four different variant call sets for strategy A: pre_trans_sam and pre_trans_gatk are the call sets generated following alignment to the transcriptome alone and pre_genome_sam and pre_genome_gatk are the corresponding files generated following whole genome alignment. Strategy B follows an identical protocol except the removal of duplicate reads post-alignment to the relevant reference. Similarly four variant call sets were generated for this strategy: post_trans_sam and post_trans_gatk following transcriptome alignment and post_genome_sam and post_genome_gatk from the genome alignments. The sensitivity and specificity of each SNP was assessed by the number of reads aligned at each individual base (providing this was greater than 3× coverage). SAMtools derived SNP calls were generated with default pileup settings and standard SNP filters. GATK derived SNP calls were filtered using standard GATK SNP filters. All calls were subsequently filtered to remove sites overlapping regions where no variant calls were attempted in the 1000 Genomes pilot study 2 [21]. SNP calls generated by SAMtools and GATK were finally filtered to remove SNP clusters, where two or more variants occurred in a three base-pair window. SNP calls passing filtering were examined for concordance with the 1000 Genomes release from March 2010 (http://browser.1000genomes.org/index.html), and dbSNP build 132.

RNA-seq reads are sampled from a gene in a manner proportional to its expression level in a given tissue. As a result, the coverage of variants varies greatly from gene to gene. In addition, mapping efficiency of individual nucleotide sites is dependent on a number of variables including regional GC content and uniqueness, and means that non-uniform base coverage across a gene or exon can occur, which will influence SNP discovery. As a consequence the definition of each gene in the transcriptome as expressed or not expressed based on median or mean base coverage per exon [26] is a poor proxy for defining the ability to call SNPs at any individual base in a gene. In this study we estimated the coverage in our sample at each site of the published 1000 Genomes variants and calculated our ability to correctly call these sites as variant in RNA-seq data at different sequencing depths. For individual NA12878, the 1000 Genomes pilot 2 study predicted a total of 2,766,610 SNPs of which 45,371 occurred within boundaries of the 23,147 genes defined as the transcriptome in this study. For NA12891, there were 2,720,364 SNPs of which 44,462 were genic and for NA12892, there were 2,736,863 SNPs of which 45,437 were genic.

### Calculation of Specificity and Sensitivity

The different metrics of RNA-seq SNP detection that we wanted to measure were the number of SNPs called per sample and the specificity and sensitivity of those SNP calls. The 1000 Genomes pilot study 2 SNP calls were used to determine the accuracy of calls made using RNA-seq data. All 1000 Genomes variant sites with > = 3X coverage in our data were treated as expected calls for this individual and used to define both the specificity and sensitivity of cDNA derived variant calls. Specificity was calculated as the number of true positives divided by the number of true positives plus the number of false positives. A true positive was any SNP present in our sample data and the corresponding 1000 Genomes and/or dbSNP data that had the correct genotype or in the case of dbSNP had the expected alleles at that position. A false positive SNP call is where the genotype in our data did not match 1000 Genomes data or the variant was not present in dbSNP. As we were dealing with transcriptome data as opposed to full genome data this meant that we will only be able to detect SNPs at sites expressed in our samples. To perform sensitivity analysis we calculated the number of true positives divided by the number of true positives plus the number of false negatives. A true positive was where we detected the expected genotype and a false negative is where we either did not detect a SNP at that position, or did detect a SNP but the genotype did not match.

SNPs identified in our RNA-seq data for which no corresponding variant was reported in the 1000 Genomes pilot study release were further examined in both dbSNP132 and the publicly available 1000 Genomes alignment (BAM) files. SNPs with a matching position and identical alternative allele call in dbSNP were considered as potentially true variants. In addition, corroborating evidence for the RNA-seq variant call was examined in the 1000 Genomes alignment files because not all true variants will have passed the filters employed in that study. For heterozygote calls we required the alternative allele to occur in between 20 and 80% of the 1000 Genomes aligned reads to provide potential evidence of a true variant occurring at a site. For homozygote non-reference calls we required >90% of reads in the 1000 Genomes alignment to carry the predicted alternative allele at a site.

## Results

The overview figure 1 demonstrates the analysis strategy that we employed to evaluate RNA-seq as a SNP detection tool. We initially applied these methods to our in-house RNA-seq data for NA12878 and the results of these analyses using all methods based on all strategies in figure 1 are detailed in table S2. We subsequently validated these methods using additional online RNA-seq data for NA12891 and NA12892, and describe these analyses at the end of the results section.

### Gene/Exon Expression in the NA12878 LCL Sample

The hg18 refFlat gene set of 23,147 unique genes is composed of 212,392 exons. Aligning 40,784,356 single-end 40 bp fragments resulted in at least one sequence read mapping to 17,014 genes and 139,143 exons. 82,091 exons had complete coverage at 1× or greater across their full length. The 23,147 genes in the refFlat gene set span 67,893,145 base pairs in the hg18 genome release. Consistently across all alignment methods employed here, 50% of these bases were not covered by a single read and 12% of the bases in the transcriptome have 1–2× coverage. The remaining 38% of sites had 3× or greater coverage and represent the total number of transcriptome spanning sites at which a genotype call was attempted in this study.

### SNP Calls

**Figure 2:**
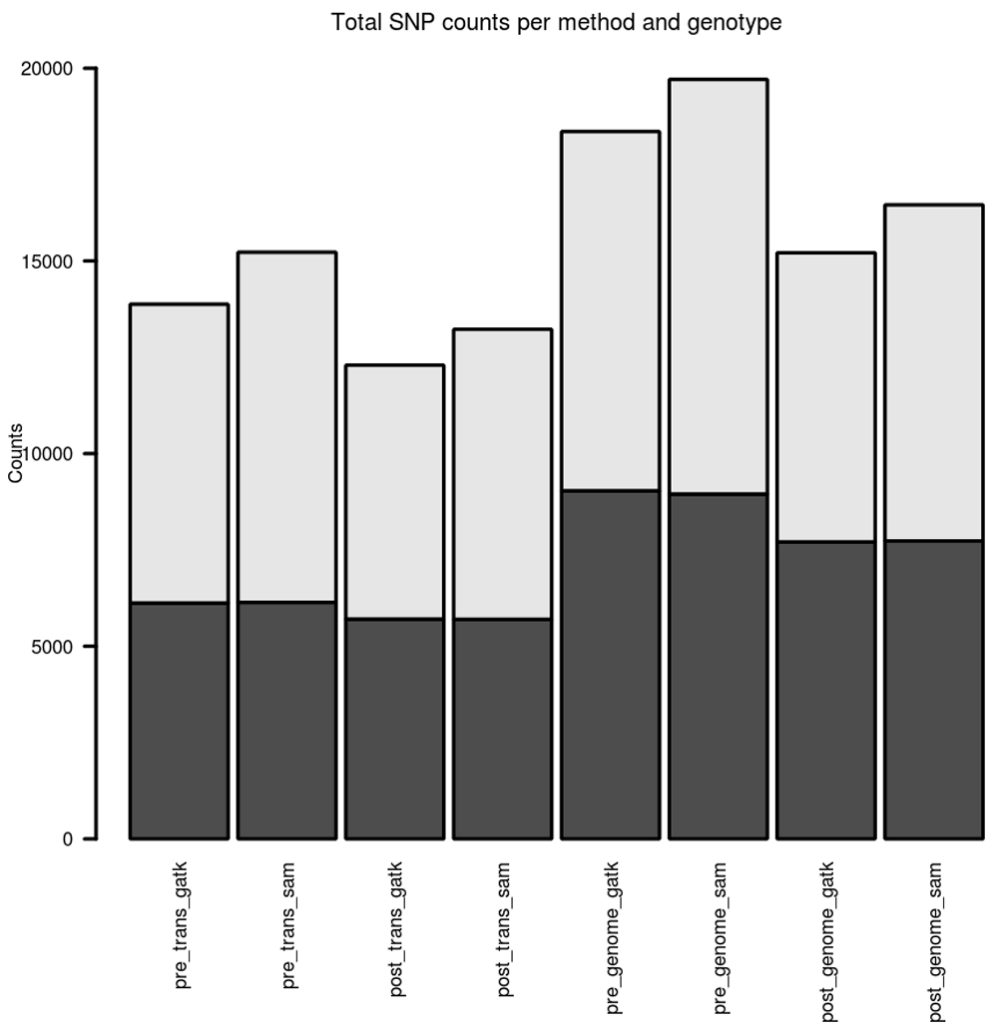
Number of SNPs per method in RNA-seq data. This figure displays the number of SNPs called for each of the 8 methods used. The proportion of heterozygous (grey) and homozygous (black) SNP calls is also displayed. Details of the numbers of SNPs called are listed in table S2a.

Figure 2 displays (a) the number of SNPs called for each of the eight methods employed for NA12878. SAMtools consistently identifies 8–10% more variants than GATK for each of the alignment methods. Approximately 96% of these extra SAMtools variants are called at coverages lower that 10× and most likely are a consequence of the differing post-calling filtering strategies employed by the two variant calling pipelines. Alignment to the transcriptome identified between 12,296 and 14,224 variants depending on the SNP caller and duplicate read removal strategy. This represents between 27 and 33% of the total expected number variants in the transcriptome of individual NA12878 based on 1000 Genomes pilot study 2. Between 80 and 88% of these called variants are identified at sites reported by the 1000 Genomes study and have an identical genotype assignment. Overall, for approximately 70% of 1000 Genomes transcriptome-overlapping SNPs a read depth of at least 3× was not generated in this study and no genotype call was attempted. On average for each transcriptome-only calling method, 50–57% of the 45,371 known sites had zero reads overlapping and represent sites in genes unexpressed in LCL or sites in expressed genes where non-uniformity of coverage across the transcript renders sites unusable. Whole genome alignment identifies between 15,213 and 19,683 SNPs depending on the method employed. The additional SNPs generated from genome alignment, when compared to the transcriptome only alignment calls, occur in regions outside those annotated as exonic in RefSeq gene annotations. Table S2 details the number of SNPs detected for the different methods at varying degrees of coverage.

### Specificity

**Figure 3:**
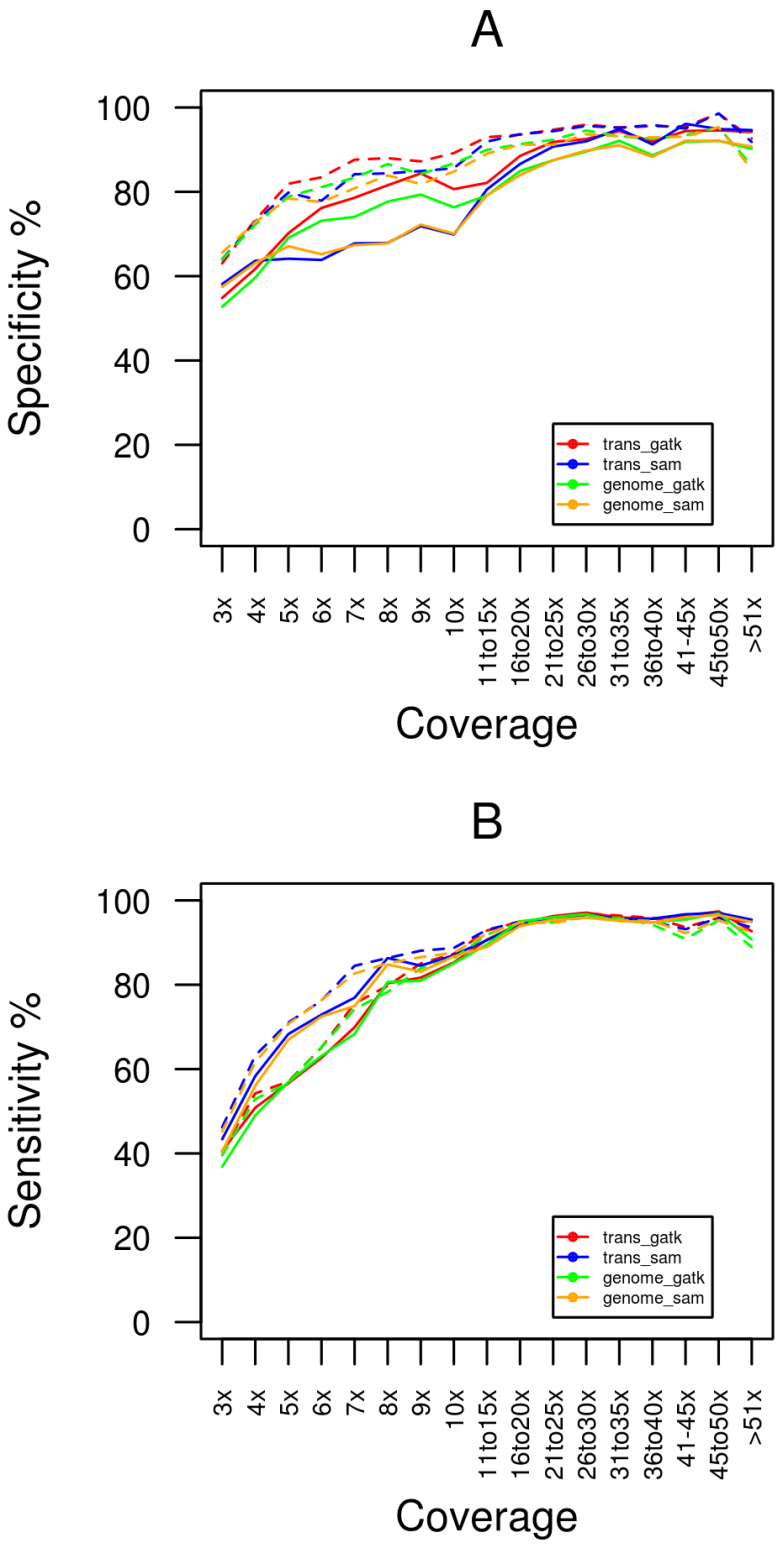
Specificity and sensitivity of the SNP calls from RNA-seq data. This figure displays the specificity (A) and sensitivity (B) of the SNP calls for each of the 8 methods at a range of coverage depths. Solid lines represent calls made where duplicate reads had been removed pre-alignment and broken lines are calls generated when duplicate reads are removed post-alignment.

**Figure 4:**
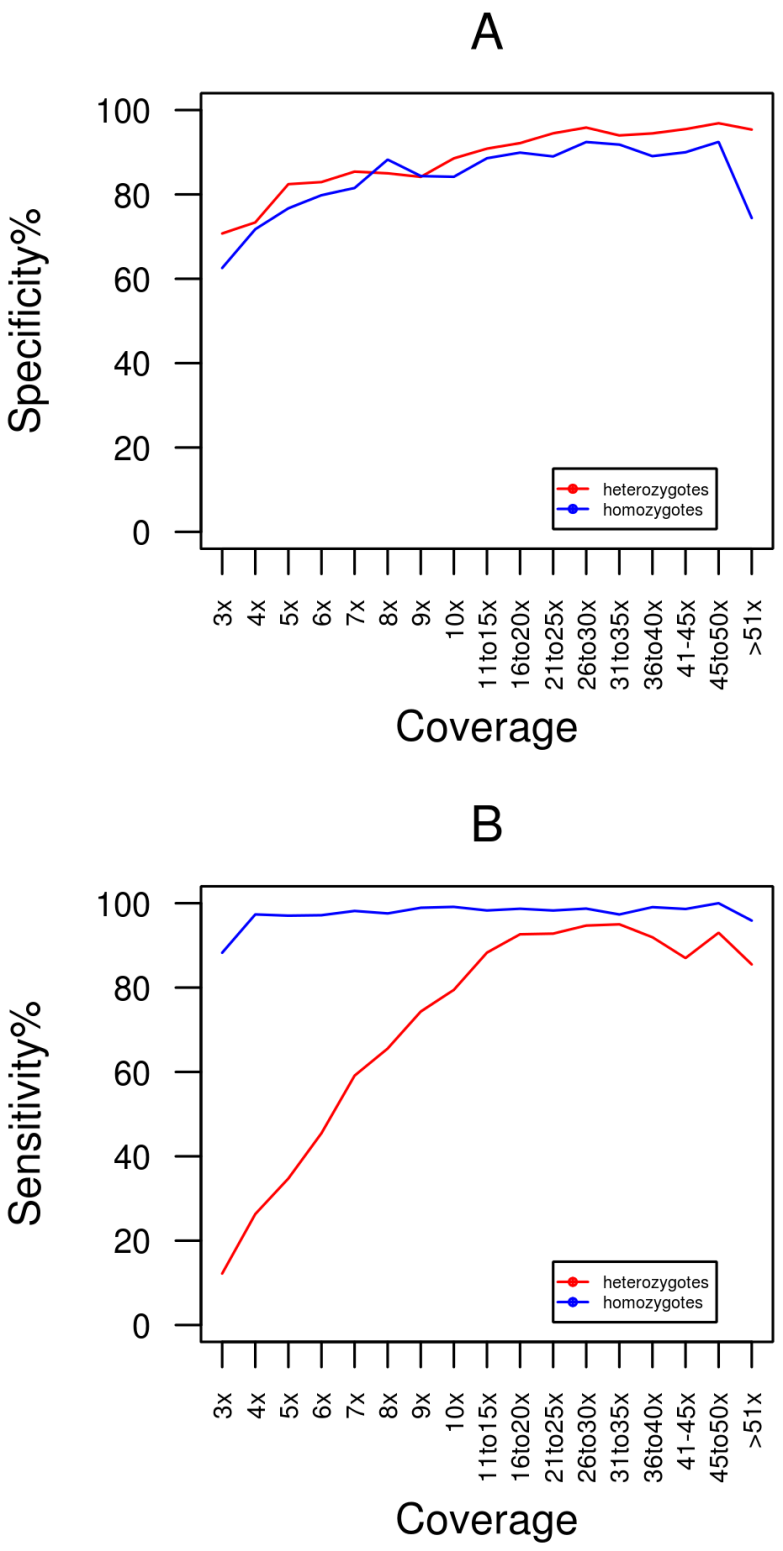
Specificity and sensitivity of heterozygous and homozygous SNP calls from RNA-seq data. This figure displays the specificity (A) and sensitivity (B) for heterozygous and homozygous SNP calls for the post_genome_gatk calling method at a range of coverage depths.

The specificity of NA12878 SNP calls based on 1000 Genomes data for each of the eight methods at varying coverage depths are displayed in figure 3A. At all read depths removal of duplicate reads post-alignment (broken lines) results in a higher degree of specificity than removal of duplicate reads pre-alignment. Even at depths as low as 3×, >60% of predicted variants represent real 1000 Genomes SNPs with specificity increasing to >90% at sites with > = 10× coverage. A consistent finding for all methods was that the specificity reached a plateau when base coverage is >10X. These data indicate that a very high proportion of SNPs detected in RNA-seq data are true variants and as expected the likelihood of an accurate SNP call increases with higher sequence coverage. The specificity of SNP calls is very similar for both heterozygous and homozygous sites (figure 4A).

For each of the calling strategies we noted a substantial number of sites which had not been identified as variant in the available 1,000 Genomes data (Table S2). Although these SNPs had been classified false positive results in our specificity analysis, some may represent variants that have not previously been recorded in analysis of this individual by 1000 Genomes as a result of insufficient coverage or being outside the employed variant filtration parameters at these particular loci in the sequence data. In order to quantify what proportion of these variants may potentially be true, we accessed the aligned BAM files from the 1000 Genomes at these loci and looked for evidence of a non-reference allele(s). The number of RNA-seq variant calls for which acceptable evidence of variation is present in the aligned 1000 Genomes data ranges between 5 and 8% indicating that a small but appreciable proportion of these variants may represent true SNPs within the our data that were not detected to date by 1,000 Genomes project analysis (Table S2). A slightly higher proportion of these SNPS are also found in dbSNP132 but most represent SNPs deposited there as part of the three 1000 genomes pilot studies (Table S2).

### Sensitivity

To perform sensitivity analysis we identified SNPs that had known genotypes from 1000 Genomes data for NA12878 and were located at sites covered by at least 3 reads in our RNA-seq data. Figure 3B shows the sensitivity for each method for the sample. Similar to the specificity analysis, at all read depths removal of duplicate reads post-alignment (broken lines) results in a higher degree of sensitivity than removal of duplicate reads pre-alignment. For all calling strategies sensitivity ranges from 40% to 80% at coverage depths below 10×. In this coverage range, sensitivity is much higher for homozygous variants compared to heterozygous variants (figure 4B). Above 10×, all methods converge at approximately 92% sensitivity (figure 3B), indicating that a very high proportion of expected variants will be detected using RNA derived reads if sufficient coverage is available.

### Extension of Analyses to NA12891 and NA12892

To investigate if the results from our analyses were reproducible when applied to other RNA-seq datasets, we applied the same methods of SNP detection to online RNA-seq data for NA12891 and NA12892. Importantly, these two samples have also been whole genome sequenced to a deep coverage by 1000 Genomes so two comprehensive sets of SNP calls are available to compare against our SNP calls from the RNA-seq data. As methods employing the removal of duplicate reads post-alignment performed best for our in-house sample, we just present data from these four methods for the two online samples.

More sequence reads were available for these two samples (80,062,322 and 80,023,151 reads for NA12891 and NA12892 respectively) compared to our in-house sample (40,784,356 reads). When aligning to transcriptome, SNP numbers are similar in all three samples for all calling methods tested (table S2a, b, c; figure S1). However, when we align to the genome, we note firstly that the results for the two on-line samples are very similar (average of 67,647 SNPs called per sample using SAMtools) but secondly we note that this SNP number is far greater than we called for our in-house sample (16,455 SNPs; figure S1). Analysis of the aligned reads indicates that the proportion of reads aligning to the annotated transcriptome is very different between our in-house sample (95%) compared to the two online samples (45%). Thus, when we align this data to the genome, the extra reads mapping off-transcriptome are generating a huge increase in SNP calls in the online sample data. This outcome could be due to differences in the sample prep protocols employed for the different samples, in particular in relation to Poly-A purification, which does influence the comparability of RNA-seq datasets [35].

**Figure 5:**
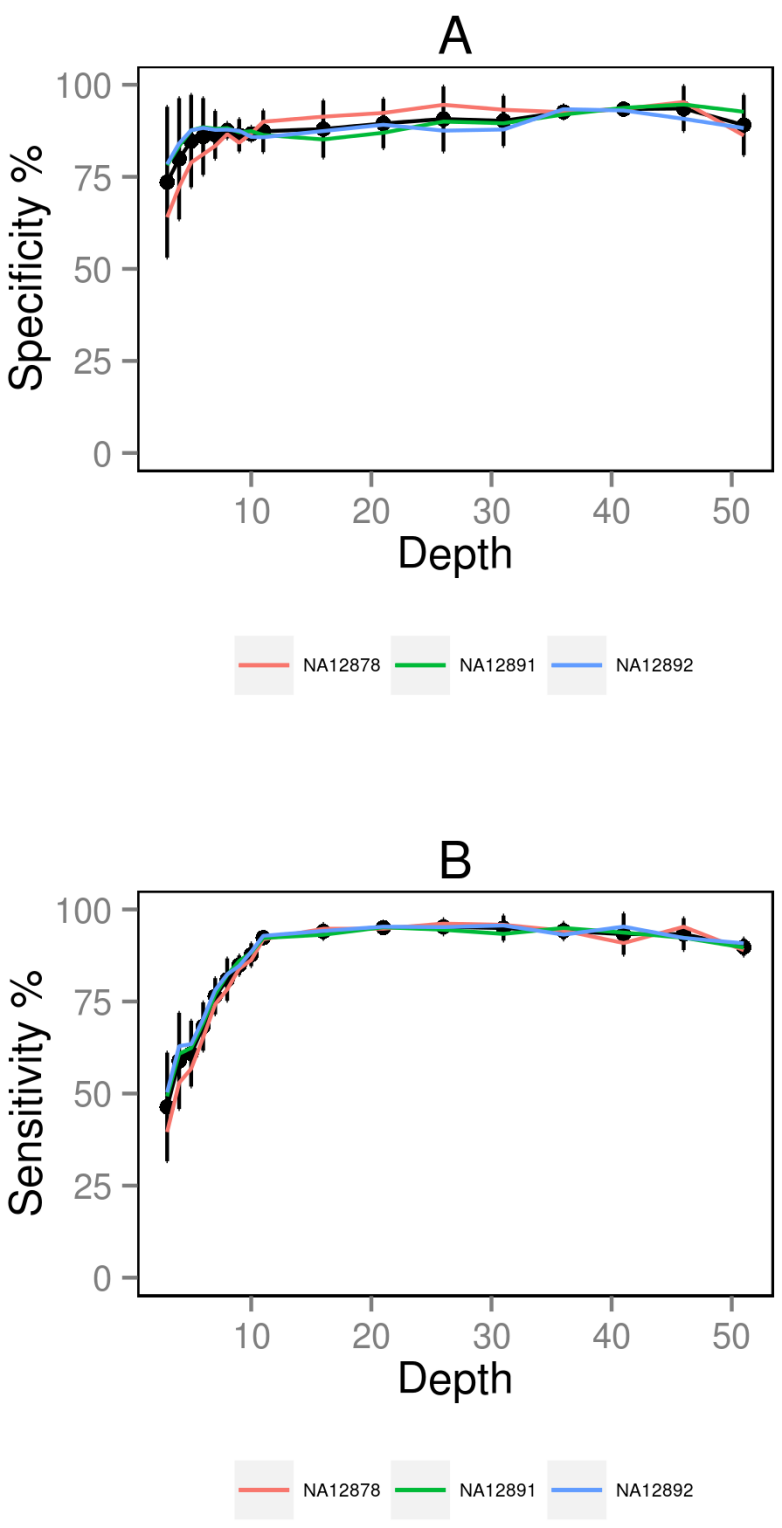
Specificity and sensitivity of the SNP calls from RNA-seq data for all three samples. This figure displays the specificity (A) and sensitivity (B) of the SNP calls for each of the three samples (in colour) at a range of coverage depths using the post_genome_sam method. The black lines plot the averages of all three samples plus 95% confidence intervals.

When we calculate specificity and sensitivity across our range of methods, we find that the results for the two online samples are near identical (figures S2, S3, S4, S5; table S2b,c). This highlights the reproducibility of the SNP calling methods when applied two RNA-seq datasets that were generated using the same methods. When removing duplicate reads post-alignment, specificity is best at all coverage levels when aligning to the genome compared to the transcriptome and there is little difference in performance between GATK and SAMtools (e.g., both 85% specificity at 10× coverage for genome). Sensitivity is marginally better for genome- compared to transcriptome-alignment and SAMtools slightly out-performs GATK at all read depths above 10× (e.g., 89% versus 88% sensitivity at 10× coverage for genome). As SAMtools is also calling more variants to begin with, it appears to be the better SNP caller for RNA-seq data. When we compare the specificity and sensitivity measurements for the two online samples to those from our in-house NA12878 sample, we do observe some differences but mostly for SNPs called at lower coverage levels (figure 5; table S2a, b, c). Above 10× coverage, the data is much less noisy, especially for the sensitivity measurements.

## Discussion

This study examined next-generation transcriptome sequencing (RNA-seq) as a method of expressed SNP detection in LCLs. Many disease samples are now biobanked as LCLs and RNA from these cell repositories are routinely used for scientific investigation. As well as data on expression and splicing, RNA-seq data can be used for SNP detection. It is therefore important to determine the best strategy for RNA-seq SNP analysis and we have addressed this question by quantifying the specificity and sensitivity of different alignment and SNP-calling methods. These results are also relevant to non-LCL derived RNA, e.g. from a different cell line type or from a disease tissue sample, because they inform on the parameters required for accurate SNP calling in RNA-seq data.

We explored different strategies for RNA-seq SNP detection by dropping duplicate sequence reads either pre- or post-alignment and using different reference data for alignment (genome and transcriptome) and different SNP-calling algorithms (SAMtools and GATK; figure 1). We note that removing duplicate reads post-alignment confers an appreciable increase in SNP detection in terms of specificity and sensitivity when compared to dropping reads pre-alignment. These differences are more pronounced at read depths below 10× and indicate how marking of PCR duplicates after alignment is more sensitive to removal of reads originating from the same genome location which contain a sequencing error rather than evidence of a true mismatching base, when compared to strategy that collapses identical reads pre-alignment.

The major difference between results of SNP-calling based on alignment to the transcriptome or the genome was the number of SNPs identified rather than the specificity or accuracy of those SNP calls. Whole genome alignment results in more calls across all methods when compared to alignment to the transcriptome alone. Specificity and sensitivity measurements are at least similar and often better for the genome alignment methods compared to the transcriptome alignment methods. Several widely used gene sets for the human genome have been generated including RefSeq (taken from the NCBI RNA reference sequences), ENSEMBL (computationally predicted from genomic sequence) and UCSC (based on protein data from Swiss-Prot/TrEMBL (UniProt) and the associated mRNA data from Genbank). Each of these gene sets contains significant differences from the others with both the RefSeq and UCSC datasets being more conservative than the ENSEMBL predictions. Even allowing for the differences between these three datasets, a significant proportion of the SNPs called following alignment to the genome occur in regions outside any annotated gene region and would be missed by any analysis using the transcriptome alone. This finding is of particular relevance to variant calling studies in organisms without a fully sequenced genome. Transcriptome *de novo* assembly followed by variant detection in such species could result in a considerable underestimation of expressed variants, unless the transcriptome is generated from reads sourced from all tissues. Sequencing to sufficient depth to completely reconstruct the transcriptome of such a species would be prohibitively expensive and with advances in sequencing technology it might be more economical to sequence the genome of such species to use as a reference for subsequent RNA related studies.

Significant overlap exists between the calls sets generated by the two SNP callers used in this study. On average 98% of GATK derived calls are found in the SAMtools call set generated on the same alignments, while approximately 91.5% of SAMtools calls overlap with those of GATK, reflecting the higher number of SNP calls by SAMtools. Comparison of results for both calling strategies employed in this study indicates that sensitivity and specificity calculations for variant calls made at > = 10× coverage using both SAMtools and GATK are virtually identical (figure 3). At coverage depths below 10×, SAMtools displays a higher degree of sensitivity but a slightly lower specificity when compared to GATK. This is because at these coverage levels, the greater number of SNPs called by SAMtools identifies a higher proportion of the expected true variants (increasing sensitivity) but with an associated increase in the number of false positive SNP calls (reducing specificity).

Cirulli et al. [26] investigated the specificity of RNA-seq as a SNP detection method by comparing whole genome and whole transcriptome sequence for one individual. RNA was sourced from peripheral blood mononuclear cells (PBMCs). When they restricted their analysis to PBMC-expressed genes; they report a specificity of 67%, which is much lower than the specificity reported here. This result was based on 8 lanes of sequence data and they report that specificity dropped as the quantity of sequence data used in the analysis increased. When this study used just one lane of sequence data (equivalent to our study), specificity was calculated as 83%; a result closer to the levels we report here. In our study we had the benefit of being able to compare our RNA-seq data to a more complete catalogue of genomic variation data, which is likely to result in more accurate specificity calculations. In addition, we utilised the masking data generated by the 1000 Genomes project to remove sites from our analysis at which variants cannot confidently be called. The addition of this step to the pipeline significantly reduces the number of false positive from the SNP call sets. In the absence of this step the specificity and sensitivity estimated for our SNP calls closely mirrors the results generated in previous studies [26]. For the three CEU individuals sequenced as part of the 1000 Genomes pilot study the “inaccessible” genome was estimated at 20% of total bases (decreasing to 15% in coding regions) [21]. The majority of these regions were removed from any variant calling analysis in that study due to difficulty in accurately mapping reads to these parts of the genome, as they mainly represent high-copy repeats or segmental duplications. Of particular relevance to RNA-seq is that more than 25% of human RefSeq genes contain at least 10% of non-unique sequence [36]. This is principally due to the high rate of gene duplication in mammalian gene families as well as the widespread presence of common functional domains amongst even non-related genes. The presence of such non-unique regions in genes has important consequences for normalisation steps in RNA-seq derived expression studies and, as shown here, using information on non-unique gene regions will help reduce the number of false positive SNP calls in RNA-seq data.

Genome mappability is influenced by both the local genomic content and the sequencing strategy employed. A recent study has produced an algorithm for determining the mappability of each base in any reference genome using a user-specified read length and mismatch number [37]. Using this method we were able to generate mappability data matching to the read length and number of sequence mismatches selected in alignment of our RNA data. SNPs excluded from our analysis using this in-house generated mappability data almost identically mirrors the sites excluded using the 1000 Genomes accessible genome information (data not shown). Use of such a mappability profile, generated with the appropriate length and mismatch criteria, must be incorporated into any RNA-seq variant calling study in order to minimise the number of spurious calls generated.

What is the cause of false positive SNP calls in our data? The majority of these discordant genotypes occur at sites of <10× coverage (figure 5). At >10× coverage, the proportion of these mis-matches drops to ∼12% for pre-alignment duplicate removal strategies and ∼6% when duplicates are removed post-alignment. For NA12878, a small number of our false positive SNP calls were in fact true variants when we examined alignment data from 1000 Genomes indicating that the exhaustive genomic sequencing of this sample may not yet have identified all variants present in this individual.

Our sensitivity analysis addressed the issue of what proportion of true variants will be detected in a RNA-seq SNP analysis. This again very much depends on coverage of the site irrespective of the overall quantity of data produced for a test sample. For all analysis methods tested here, overall sensitivity is >90% when there is >10X coverage but can drop as low as 40% if a site has 3× coverage. As expected, the majority of false negative sites in our RNA-seq sample occur at heterozygous sites where we have low coverage (figure 4B). At such sites sufficient evidence for the alternative allele may not be present in order to confidently emit a non-reference call. The reduction of the false-negative rate to ∼5% above 10× coverage suggests that sequencing to a higher depth should decrease this rate appreciably.

There are a number of additional factors that affect both sensitivity and specificity. All calculations in this study are based on 1000 Genomes data being correct. Comparison of available HapMap and 1000 Genomes data for sample NA12878 identified 1,964,991 SNPs common to both datasets; however genotypes did not match for 2% of these SNPs. Other factors to consider are errors in the RNA-seq data (sequencing errors or artefacts of the EBV cell line transformation), allelic imbalance in the RNA, random mono-allelic expression in clonal cell lines [38], or instances of RNA editing. The majority of sequencing errors should be filtered out providing the sequence/base quality is high enough but it is not possible to exclude all errors. Allelic expression differences in the RNA whereby one allele at an expected heterozygous site is over-expressed and appears as a homozygote would result in a mis-match genotype call between DNA and RNA. RNA-seq has been used in several studies for the identification of these sites[7], [39]–[41]. RNA editing is a post-transcriptional mechanism of base re-coding through insertion, deletion or modification of nucleotides and has been associated with a number of diseases, including several neurological disorders [42], [43]. We do not believe RNA editing to have a major influence on SNP calling in our study because only 0.4% of our false negative calls occurs at known sites of RNA editing ([44]; data not shown).

We have shown that accurate variant detection is possible using cDNA derived sequencing reads at sites with >10× coverage. In our study of a LCL sample, SNP calling was possible at 38% of the annotated transcriptome. The prohibitive cost associated with sequencing to the required depth, to accurately call all expressed variants in a sample, means that while RNA-seq variant calling works well, it is not a viable alternative to exome or whole genome sequencing. Use of longer and/or paired-end reads will increase the base coverage within expressed genes in a sample and as a result increase the sensitivity of the SNP calls generated, but this will be limited to SNPs occurring in relatively highly expressed genes.

In conclusion, SNP calls in RNA-seq data is a useful by-product of the technique and boosts the amount of data that can be generated from such experiments and potentially used in convergent functional genomics research. We found that in addition to the computational benefit of reducing the number of reads to be processed in downstream steps, removing duplicate sequences post-alignment will increase the quality SNPs called overall. We found that aligning sequence reads to a genomic rather than transcriptomic reference will increase the number of SNPs called, with the additional SNPs occurring at bases not currently curated as genic regions in human genome annotation sets and greater specificity is achieved when aligning to the genome. SAMtools identifies 8–10% more variants that GATK and thus detects more true SNPs than GATK (higher sensitivity) but also calls more false positives than GATK at low coverage (lower specificity). When we applied our methods to multiple RNA-seq datasets that had being prepared and sequenced with the same methods (online samples NA12891 and NA12892), the results for metrics of SNP detection were very reproducible. When we compared these data to our original in-house sample (NA12878), the main difference was the overall number of SNPs detected. This indicated that the number of sequence reads used in an analysis and original preparation of the sequencing library will influence SNP detection. Despite this large difference in SNP number, the only differences of note between the samples were found at lower coverages. At >10× coverage, specificity and sensitivity were very similar, again highlighting the reproducibility of the methods. Overall, using appropriate methods and filters, a very high proportion of SNPs called in RNA-seq data will be true variants and RNA-seq SNP analysis will identify the majority of variants present in expressed exons provided sufficient coverage is available.

## Supporting Information

Figure S1 **Number of SNPs per method in RNA-seq data.**
(PDF)

Figure S2 **Specificity of the SNP calls from RNA-seq data for NA12891.**
(PDF)

Figure S3 **Specificity of the SNP calls from RNA-seq data for NA12892.**
(PDF)

Figure S4 **Sensitivity of the SNP calls from RNA-seq data for NA12891.**
(PDF)

Figure S5 **Sensitivity of the SNP calls from RNA-seq data for NA12892.**
(PDF)

Table S1 **Number of sequence reads per sample per method.**
(XLS)

Table S2 **Analysis results for all three RNA-seq sample datasets.**
(XLS)
