# Supplementary material for: Development of Strategies for SNP Detection in RNA-Seq Data: Application to Lymphoblastoid Cell Lines and Evaluation Using 1000 Genomes Data
Source: PLoS One. 2013 Mar 26;8(3):e58815. doi: 10.1371/journal.pone.0058815 (PMC3608647; doi:10.1371/journal.pone.0058815)
Supplement: Figure S1 — Number of SNPs per method in RNA-seq data. (PDF) [file pone.0058815.s001.pdf]

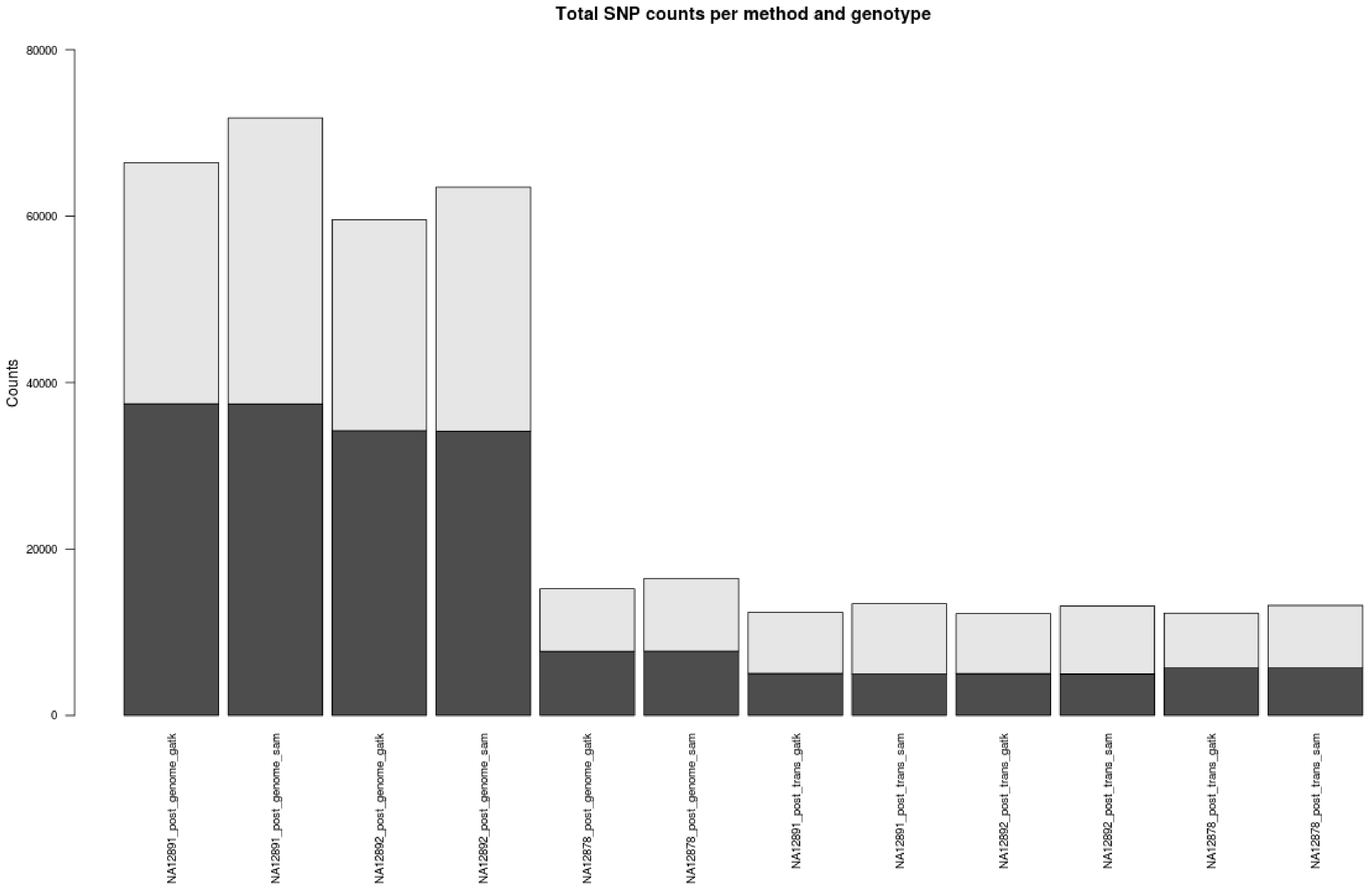

Supplementary Figure 1: Number of SNPs per method in RNA-seq data. This figure displays the number of SNPs called for each of the 3 samples using 4 different methods. The proportion of heterozygous (grey) and homozygous (black) SNP calls is also displayed. Details of the numbers of SNPs called are listed in supplementary table S2a-c.
