# Supplementary material for: Development of Strategies for SNP Detection in RNA-Seq Data: Application to Lymphoblastoid Cell Lines and Evaluation Using 1000 Genomes Data
Source: PLoS One. 2013 Mar 26;8(3):e58815. doi: 10.1371/journal.pone.0058815 (PMC3608647; doi:10.1371/journal.pone.0058815)
Supplement: Figure S5 — Sensitivity of the SNP calls from RNA-seq data for NA12892. (PDF) [file pone.0058815.s005.pdf]

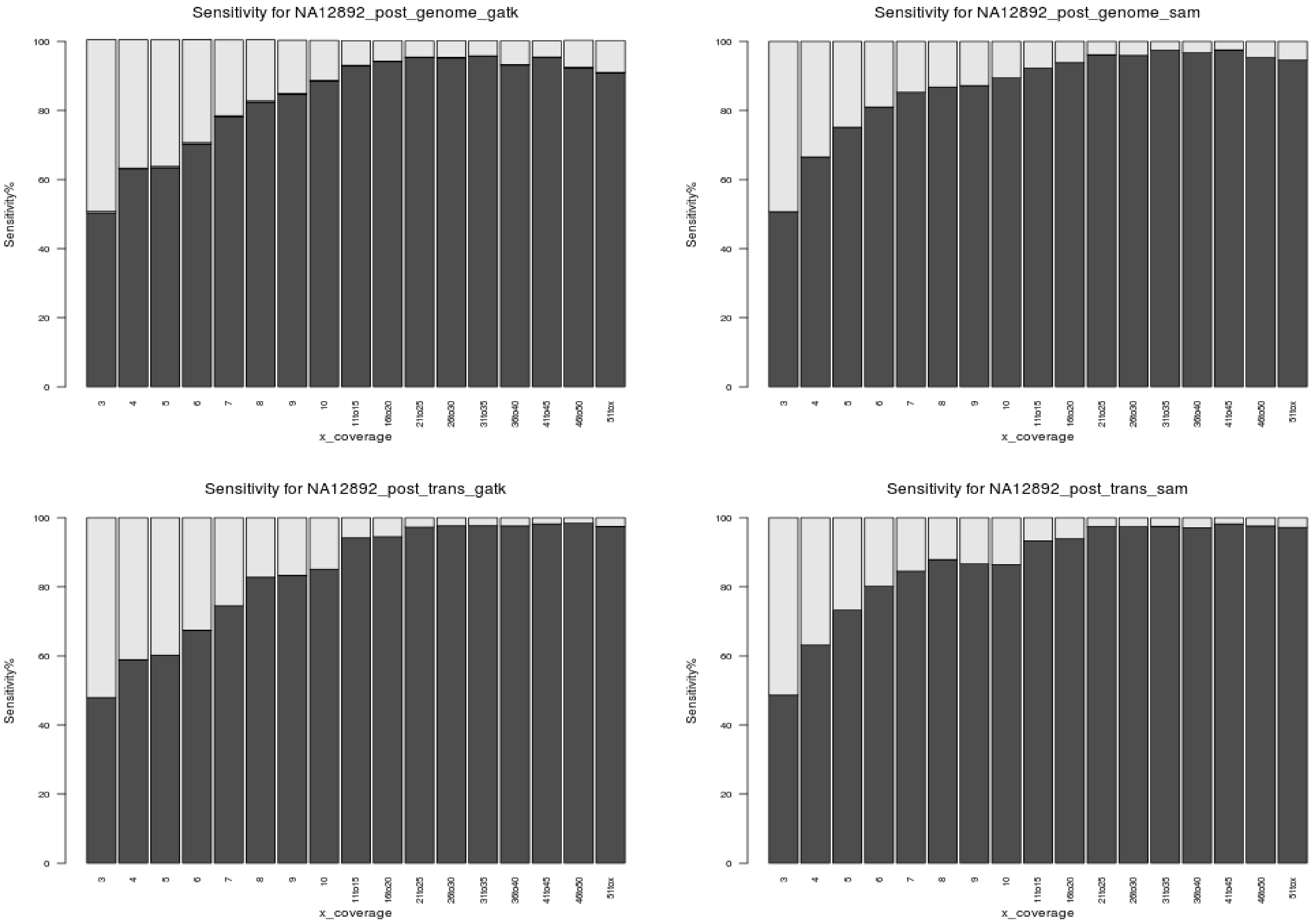

Supplementary Figure 5: Sensitivity of the SNP calls from RNA-seq data for NA12892. This figure displays the sensitivity of the SNP calls for NA12892 using 4 methods at a range of coverage depths.
